# *Mycoplasma hyopneumoniae* Infection Activates the NOD1 Signaling Pathway to Modulate Inflammation

**DOI:** 10.3389/fcimb.2022.927840

**Published:** 2022-07-08

**Authors:** Wei Liu, Pengcheng Jiang, Keli Yang, Qiqi Song, Fangyan Yuan, Zewen Liu, Ting Gao, Danna Zhou, Rui Guo, Chang Li, Pei Sun, Yongxiang Tian

**Affiliations:** ^1^ Key Laboratory of Prevention and Control Agents for Animal Bacteriosis (Ministry of Agriculture and Rural Affairs), Hubei Provincial Key Laboratory of Animal Pathogenic Microbiology, Institute of Animal Husbandry and Veterinary Sciences, Hubei Academy of Agricultural Sciences, Wuhan, China; ^2^ Anhui Province Key Laboratory of Veterinary Pathobiology and Disease Control, College of Animal Science and Technology, Anhui Agricultural University, Hefei, China; ^3^ Tianjin Key Laboratory of Agricultural Animal Breeding and Healthy Husbandry, College of Animal Science and Veterinary Medicine, Tianjin Agricultural University, Tianjin, China

**Keywords:** *Mycoplasma hyopneumoniae*, mhp390, NOD1, inflammation, interaction.

## Abstract

*Mycoplasma hyopneumoniae* is a highly contagious pathogen causing porcine enzootic pneumonia, which elicits prolonged inflammatory response modulated by pattern recognition receptors (PRRs). Although significant advances have been achieved in understanding the Toll-Like receptors that recognize *M. hyopneumoniae*, the role of nucleotide-binding oligomerization domain 1 (NOD1) in *M. hyopneumoniae* infected cells remains poorly understood. This study revealed that *M. hyopneumoniae* activates the NOD1-RIP2 pathway and is co-localized with host NOD1 during infection. siRNA knockdown of NOD1 significantly impaired the TRIF and MYD88 pathway and blocked the activation of TNF-α. In contrast, NOD1 overexpression significantly suppressed *M. hyopneumoniae* proliferation. Furthermore, we for the first time investigated the interaction between *M. hyopneumoniae* mhp390 and NOD1 receptor, and the results suggested that mhp390 and NOD1 are possibly involved in the recognition of *M. hyopneumoniae.* These findings may improve our understanding of the interaction between PRRs and *M. hyopneumoniae* and the function of NOD1 in host defense against *M. hyopneumoniae* infection.

## Introduction

Germline-encoded pattern recognition receptors (PRRs) can sense and rapidly respond to structures derived from distinct bacteria and viruses, which is known as the pathogen-associated molecular patterns (PAMPs) (Takeuchi and Akira, 2010). To date, four main PRR families have been documented, including Toll-like receptors (TLRs), retinoic acid-inducible gene (RIG)-I-like receptors (RLRs), C-type lectin receptors (CLRs) and NOD-like receptors (NLRs) (Walsh et al., 2013). TLRs and RLRs can recognize structurally conserved molecules derived from microbes and play a key role in the innate immune system (Lupfer et al., 2013). NLRs can trigger multiple signaling cascades such as the proinflammatory nuclear factor B (NF-κB) and the inflammasome pathways, as well as autophagy and cell death pathways (Philpott and Girardin, 2010). Nucleotide-binding oligomerization domain 1 (NOD1) and NOD2 are the most extensively studied members of the NLR family, which are responsible for triggering the host immune response (Murillo et al., 2003; Tourlomousis et al., 2020; Yoo et al., 2021).

Notable progress has been made in understanding the critical role of NLRs in eliciting immune responses against bacteria and virus (Moreira and Zamboni, 2012; McKernan, 2020). Previous studies have revealed a critical role of NOD1 in innate immune response and inflammatory regulation during infection by *Pseudomonas aeruginosa* (Travassos et al., 2005), *Streptococcus pneumoniae* (Opitz et al., 2004) and other intracellular bacteria such as *Shigella flexneri* (Girardin et al., 2001) or *Listeria monocytogenes* (Opitz et al., 2006). More recently, a number of studies have demonstrated that both RNA viruses (from the families of Orthomyxoviridae, Rhabdoviridae, Paramyxioviridae, Flaviviridae, and Picornaviridae) and DNA viruses (from the families of Poxviridae, Adenoviridae, Herpesviridae) can activate the NLRP3 inflammasome pathway (Lupfer and Kanneganti, 2013; da Costa et al., 2019; Boal-Carvalho et al., 2020; Choudhury et al., 2021). Inflammasomes are cytosolic multiprotein oligomers of the innate immune system, which are responsible for activating the inflammatory response. Moreover, NOD1 was shown to participate in the triggering of innate immunity signaling by Hepatitis C virus (Vegna et al., 2016) and be associated with the susceptibility of ducks to Duck Hepatitis A virus (Liang et al., 2021).

Porcine enzootic pneumonia (EP), which is caused by *Mycoplasma hyopneumoniae* (*M. hyopneumoniae*, Mhp), leads to persistent dry cough and chronic pneumonia in swine (Liu et al., 2011). *M. hyopneumoniae* is highly contagious and widely perceived as an important element of the porcine respiratory disease complex (PRDC), which causes significant economic losses to the global swine industry (Minion et al., 2004). This pathogen can destroy the ciliary barrier and cause immune suppression, increasing the possibility of secondary infection and severity of clinical symptoms (Bai et al., 2013; Liu et al., 2017; Nueangphuet et al., 2021). *M. hyopneumoniae* infection can also stimulate the host signal transduction pathway and induce the release of pro-inflammatory cytokines such as IL-1β, IL-6, IL-8 and TNF-α (Chae, 2016; Eddicks et al., 2021). Highly virulent *M. hyopneumoniae* strains have a higher proliferation titer in the lungs and induce more severe inflammation than lowly virulent strains (Meyns et al., 2007). TLR2 and TLR4 play crucial roles in mediating the IgA response induced by *M. hyopneumoniae* (Li et al., 2019). Treatment with anti-porcine TLR2 and TLR6 antibodies blocked the production of TNF-α from *M. hyopneumoniae* infected macrophages, indicating the involvement of TLR2/TLR6 in the recognition of *M. hyopneumoniae* (Okusawa et al., 2004). Although research on TLRs has greatly improved our understanding of this pathogen, the contribution of NOD1 to *M. hyopneumoniae* infection remains largely unknown. Clarification of the interaction between the pathogen and the host NOD1 can help to further elucidate the pathogenic mechanism of *M. hyopneumoniae*.

This study explored the expression dynamics of NOD1 upon *M. hyopneumoniae* infection, and revealed that *M. hyopneumoniae* activates the NOD1-RIP2 pathway to induce inflammatory cytokine production and therefore inhibit *M. hyopneumoniae* proliferation. We also demonstrated the interaction between *M. hyopneumoniae* adhesin mhp390 and NOD1 receptor, and the results indicated that they are possibly involved in the recognition of *M. hyopneumoniae.*

## Materials and Methods

### Cells, Bacterial Strains, Growth Conditions and Regents

Porcine alveolar macrophages (PAM, swine 3D4/21 cell lines) were cultured in Roswell Park Memorial Institute 1640 Medium (RPMI 1640) supplemented with 10% heat-inactivated fetal bovine serum (FBS), 100 U/mL penicillin, and 100 μg/mL streptomycin at 37°C in a humidified 5% CO_2_ incubator. The medium used for maintenance of PAMs was RPMI-1640 (Life Technology, USA) supplemented with 2% heated-inactivated FBS, 100 U/mL penicillin, and 100 μg/mL streptomycin. *Escherichia coli* strains DH5a and BL21 (DE3) were cultured in Luria Bertani (LB) medium supplemented with ampicillin (100 mg/mL) or kanamycin (50 mg/mL) when needed. *M. hyopneumoniae* strain 168 was isolated from the Er-hua-nian pig with typical clinical and pathogenic characteristics of mycoplasmal pneumonia of swine (Liu et al., 2011), which was kindly provided by Guoqing Shao (Jiangsu Academy of Agricultural Sciences, Nanjing, China). The strain 168 was grown in KM_2_ cell-free medium (modified Friis’ medium supplemented with 20% pig serum) in a humidified 5% CO_2_ incubator at 37°C.

### Plasmids and Antibodies

The P68 (pET30a-mhp390) and P97 expression plasmids were prepared in our laboratory as described previously (Liu et al., 2019b). The cDNA of NOD1 was cloned from swine PK15 cells and inserted into pCMV-HA vector (Clontech) to generate HA-NOD1 expression plasmid (*Eco*RI and *Xho*I restriction sites, primers listed in **Table S1**), which expressed HA-tagged proteins. The constructed plasmids were verified by nucleotide sequencing analysis. The commercial antibodies used in this study included polyclonal antibody against LC3 purchased from Proteintech (Cat No. 14600-1-AP, China). NOD1 monoclonal antibody was purchased from Santa Cruz (Cat No. sc-398696, USA). Anti-β-actin was obtained from Beyotime (Cat No. AF0003, China). Anti-mhp390 or anti-mhp384 polyclonal antibody was produced in rabbits through immunization with mhp390 or mhp384 protein respectively in our laboratory (Liu et al., 2019b).

### Expression and Purification of mhp390 and NOD1

The plasmids pET30a-mhp390 and pET30a-NOD1 (*Eco*RI and *Xho*I restriction sites, primers listed in **Table S1**) were transformed into *E. coli* BL21 (DE3) to express the recombinant protein. The resultant recombinant protein tagged with 6×His was purified with Ni-NTA agarose (Bio-Rad, Shanghai, China). The concentration of the purified protein was measured by using the BCA Protein Assay Kit (Beyotime, China).

### RNA Exaction and Quantitative Real-Time PCR

PAM cells grown in 6-well plates (Corning, Inc., USA) were infected with *M. hyopneumoniae* (10^7^ Color Change Unit, CCU) or mock-infected. Total RNA was isolated at the indicated time points using the TRIzol reagent (Invitrogen). Then, the RNA was reverse transcribed into cDNA through TransScript One Step RT-PCR SuperMix (Trans, Beijing, China). qRT-PCR was performed using PerfectStart green qPCR super Mix (TransGen Biotech, China) in the LightCycler 96 (Roche Molecular Biochemicals). The PCR conditions were as follows: pre-denaturing at 95°C for 5 min, denaturing at 95°C for 15 s, annealing at 58°C for 30 s, extension at 72°C for 20 s, and a total of 40 cycles of amplification. The fold change in gene expression relative to the normal was calculated using the delta delta cycles to threshold (ΔΔCt) method. Primers (**Table S1**) were designed with the Primer Express software (version 3.0; Applied Biosystems, CA). The experiments were performed independently for three times.

### Transfection

Transient transfection was performed by using Lipofectamine 2000 (Invitrogen). PAM cells were seeded on 6/12-well plates and cultured until the cells reached approximately 70%–80% confluence, and then transfected with the indicated plasmids or siRNA. For each transfection, 3 ng of the HA-NOD1 expression plasmid or 40 pM of siRNA was used. Cell extract was collected at 6, 9, 12, and 24 h post infection (hpi).

### siRNA-Mediated Interference of NOD1

The siRNA targeting porcine NOD1 or negative control siRNA was synthesized by GenePharma (China). Knockdown of endogenous NOD1 in PAM cells was carried out by transfection of NOD1 siRNA. Transient transfection of siRNA was performed by using lipofectamine 2000 (Vazyme, China) according to the manufacturer’s instructions. The siRNA sequences used were as follows: si2815-NOD1, sense 5’-CAGACGUUGAAACACUUAUTT-3’, antisense 5’-AUAAGUGUUUCAACGUCUGTT-3’; Negative control, sense 5’-UUCUUCGAACGUGUCACGUTT-3’, antisense 5’-ACGUGACACGUUCGG AGAATT-3’.

### Western Blot Assay

Cell extracts were prepared by adding 200 μL 2 × lysis buffer A (LBA) (65 mM Tris–HCl [pH 6.8], 4% sodium dodecyl sulfate, 3% DL-dithiothreitol, and 40% glycerol) and sonicated. The extracts were lysed by boiling in sodium dodecylsulphate (SDS) sample buffer (2% SDS, 60 mM Tris-HCl [pH 6.8], 10% glycerol, 0.001% bromophenolblue, and 0.33% β-mercaptoethanol), and then loaded on acrylamide sodium dodecyl sulphate polyacrylamide (SDS-PAGE) gel. The separated proteins were transferred onto PVDF membranes (Millipore, Billerica, MA). The resultant membranes were blocked with 5% (w/v) dried skim milk in tris-buffered saline containing 0.05% (v/v) Tween 20 (TBST) and incubated with primary antibodies. The antibodies used in western blot assay were anti-NOD1 monoclonal antibody (Santa Cruz, USA), and anti-mhp384 polyclonal antibody (Liu et al., 2019b). β-actin was detected with an anti-beta-actin monoclonal antibody (MAb) (Beyotime, China) to serve as the loading control. Secondary antibodies, horseradish peroxidase-conjugated anti-mouse and anti-rabbit IgG antibody, were used at a dilution of 1:4000, and the protein bands were visualized through enhanced chemiluminescence (ECL) reagents (Thermo, USA) following the manufacturer’s instructions. The signal was acquired by scanning the membranes, while the number of pixels per surface area was quantified by using ImageJ software.

### Flow Cytometry Analysis

The role of mhp390 in *M. hyopneumoniae* adherence was evaluated through flow cytometry. PAM cells were seeded in 24-well plates, and then rMhp390 was incubated with PAM cells at 37°C for 30 min. Each well was washed for 5 times to remove the unbound protein. The bound rMhp390 was labeled green through anti His-tag mouse mAb and goat anti-mouse IgG-FITC. *M. hyopneumoniae* adhesin P97R1 and BSA served as the positive and negative control, respectively. The fluorescence intensity of PAM cells was evaluated with flow cytometry (BD C6 plus flow cytometer).

For the rMhp390 inhibition assay, *M. hyopneumoniae* was pre-blocked with two-fold serial dilution of rMhp390, which corresponded to 1.25–10.0 μg/ml of protein. BSA (10 μg/mL) and 1 mL of RPMI 1640 alone were used as the negative and blank controls, respectively. For the rMhp390 antiserum inhibition assay, *M. hyopneumoniae* was incubated with anti-rMhp390 serum diluted from 1:50 to 1:1000 before infection. The mixed negative serum (unimmunized rabbit serum, 1:50 dilution) was used as the negative control. The cells were evaluated with flow cytometry (BD C6 plus flow cytometer) and the adhesion ability of *M. hyopneumoniae* was evaluated based on the fluorescence intensity of PAM cells. These experiments were performed independently for three times.

### Confocal Microscopy Assay

PAM cells were propagated in RPMI-1640 on microscope coverslips in 6-well plates (Corning, Inc., USA). *M. hyopneumoniae* was cultured in a 5% CO_2_ atmosphere at 37°C for 42 h and fluorescently labeled by incubation with 5 μM CFDA-SE at 37°C for 30 min (avoid light). The bacteria were washed three times to remove excess CFDA-SE. PAM cells (10^6^) were treated with fluorescently labeled *M. hyopneumoniae* (10^8^ CCU) or PBS at 37°C. The cells were washed with PBS for three times and fixed with 4% neutralized paraformaldehyde for 15 min at room temperature. Then, the cells were blocked with 1% (w/v) BSA in PBS (pH 7.4) overnight at 4°C. Endogenous NOD1 was detected by incubation with mouse anti-NOD1 mAb (Santa Cruz, USA) at 37°C for 1 h, and then overlain with Cy3-conjugated affinipure goat anti-mouse IgG (H+L) diluted at 1:100 (Proteintech, China) at 37°C for 1 h. 4,6-Diamidino-2-phenylindole (DAPI) (Beyotime, China) was used to label the cell nuclei. The cells were washed with PBS between each step. Immunofluorescence was detected with a Zeiss LSM900 laser scanning confocal microscope (Zeiss LSM 900 with Airyscan 2, Germany).

### Biolayer Interferometry Assay

The binding kinetics of rMhp390 and rNOD1 was monitored with a biolayer interferometry on ForteBio Octet Red96 instrument (ForteBio, USA). rMhp390 was labeled with biotin through LinKine™ biotin labeling kit (Abbkine, China) and immobilized on the streptavidin (SA) biosensors. After equilibrium in PBST (PBS containing 0.02% (v/v) Tween20) for 300 s, association between rMhp390 and rNOD1 was performed for 300 s. Dissociation was carried out in PBST buffer for 600 s. The kinetic dissociation rate constant (K_D_) was obtained using a 1:1 binding model. The association and dissociation constants were obtained using the DataAnalysisHT software (ForteBio, USA) version 12.0.

### Statistical Analysis

Statistical analysis was performed with the two-way analysis of variance test using the GraphPad PRISM software (version 8.4.3 for Windows; www.graphpad.com/) to evaluate the potential differences among different groups. Data were presented as mean ± standard deviation (SD). P-value < 0.05 was considered as significant; P-value < 0.01 was considered as highly significant; P-value < 0.001 was considered as extremely significant; and ns represents no difference.

## Results

### *M. hyopneumoniae* Infection Steadily Up-Regulates the Expression of NOD1

**Figure 1:**
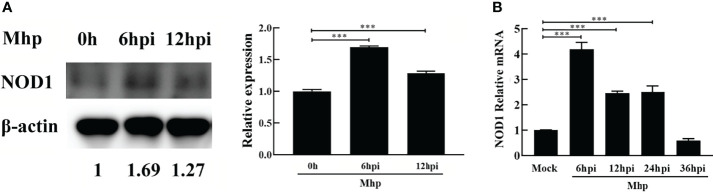
*M. hyopneumoniae* infection triggers NOD1 transcription and steadily up-regulates NOD1 protein abundance. **(A)** *M. hyopneumoniae* infection up-regulates NOD1 protein abundance. **(B)** *M. hyopneumoniae* infection triggers NOD1 transcription. ***P < 0.001 indicate statistically significant differences among different groups.

PAM cells are highly permissive for *M. hyopneumoniae* infection (Bai et al., 2013; Liu et al., 2017). To investigate the expression kinetics of NOD1 after challenge with *M. hyopneumoniae*, PAM cells were infected with *M. hyopneumoniae* (100 μL, 10^8^ CCU). The infected cells were collected at 0, 6, 12, 24, 36 hpi, and then subjected to western blotting and qRT-PCR using the primers listed in **Table S1**. As shown in **Figure 1A**, *M. hyopneumoniae* infection steadily increased the expression of NOD1 protein to nearly 1.69 folds at 6 hpi. A similar increase in the mRNA expression of NOD1 was observed after *M. hyopneumoniae* infection, with a maximum level of 4.19 folds at 6 hpi (**Figure 1B**).

### *M. hyopneumoniae* Employs Host NOD1 to Activate the Innate Immune Signaling Pathway

**Figure 2:**
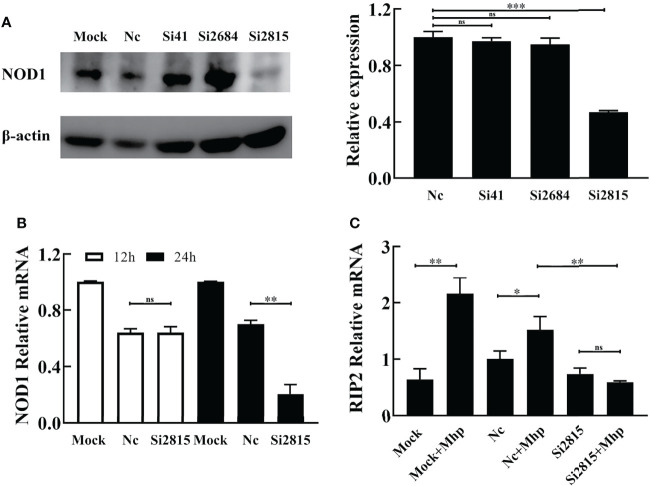
Knockdown of NOD1 inhibits the activation of RIP2 signaling pathway by *M. hyopneumoniae*. **(A)** Silencing efficiency of siRNA targeting NOD1. Alveolar macrophages were transfected with the indicated siRNA. The expression of NOD1 was evaluated by western blot at 24 h post transfection. Anti-β-actin was included as a control for sample loading. The relative levels of NOD1 in comparison to those in NC siRNA treated cells are shown as folds in the right panel. **(B)** The mRNA transcription of NOD1 was measured through real time RT-PCR and compared with that of GAPDH at 12 h/24 h post infection (hpi). **(C)** Alveolar macrophages were transfected with NOD1 siRNA or NC siRNA for 24 h, and then infected with *M. hyopneumoniae* (100 μL, 10^8^ CCU). Cells were collected at 6 hpi, and subjected to real-time RT-PCR to determine the expression of RIP2 mRNA. *P < 0.05, **P < 0.01, and ***P < 0.001 indicate statistically significant differences among different groups, and ns represents no difference.

siRNA-mediated interference was performed to determine whether the silencing of NOD1 affects the *M. hyopneumoniae*-induced innate immune signal pathway. As shown in **Figure 2A**, the knockdown efficiency of the NOD1-specific si2815 was over 50% at the protein level, while the mRNA knockdown efficiency of si2815 was about 30% at 24 h post transfection (hpt) (**Figure 2B**). No obvious interference of NOD1 was found by using si41 or si2684. Thus, NOD1 si2815 was used for further experiments in this study.

It is well known that the cytosolic receptor NOD1 can sense bacterial peptidoglycan and activate the NF-κB signaling pathway through its adaptor protein, the receptor-interacting protein 2 (RIP2), which will lead to the production of multiple inflammatory cytokines (Jing et al., 2014). Therefore, we investigated whether interference of NOD1 blocks the activation of RIP2 expression in PAM cells upon *M. hyopneumoniae* infection. As shown in **Figure 2C**, the mRNA of RIP2 was dramatically decreased in NOD1 siRNA-treated cells compared with that in the NC siRNA cells at 12 hpi.

**Figure 3:**
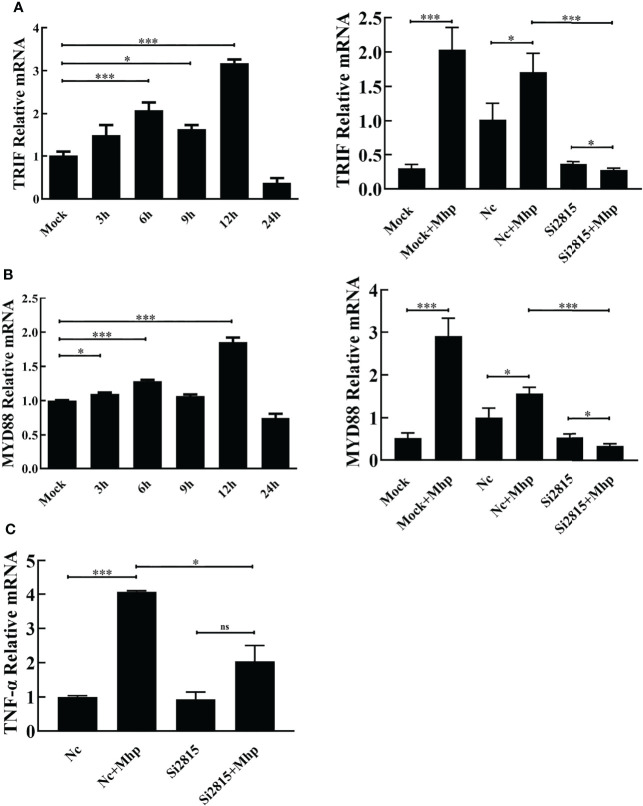
*M. hyopneumoniae* infection activates NOD1-mediated TRIF and MYD88 signaling pathways. *M. hyopneumoniae* infection induces TRIF **(A)** and MYD88 **(B)** transcription in a time-dependent manner. Alveolar macrophages were infected with *M. hyopneumoniae* (100 μL, 10^8^ CCU) or mock-infected. Cells were collected separately at the indicated time points, and subjected to qRT-PCR to analyze the relative mRNA transcription. Alveolar macrophages were transfected with NOD1 siRNA or NC siRNA for 24 h, and the cells were then mock infected or infected with *M. hyopneumoniae* (100 μL, 10^8^ CCU). The cells were collected at 12 hpi. The MYD88 and TRIF mRNA expression levels were determined by qRT-PCR assay. **(C)** Knockdown of NOD1 blocked *M. hyopneumoniae*-induced mRNA expression of TNF-α. Transfection and infection experiments performed as described for panels **(A, B)** The cells were collected at 12 hpi and subjected to qRT-PCR to determine the mRNA expression. *P < 0.05, and ***P < 0.001 indicate statistically significant differences among different groups, and ns represents no difference.

To study the involvement of the NOD1-mediated innate immune signaling pathway in *M. hyopneumoniae* infection, we firstly determined the mRNA levels of pro-inflammatory cytokines in PAMs after *M. hyopneumoniae* infection by qRT-PCR using primers listed in **Table S1**. As shown in **Figures 3A, B**, the mRNA expression of both TRIF and MYD88 was significantly up-regulated with a maximum increase at 12 hpi. In addition, *M. hyopneumoniae* infection remarkably enhanced the mRNA expression of TNF-α (**Figure 3C**).

Then, PAM cells were transfected with si2815 or the negative control (NC). At 24 hpt, the transfected cells were stimulated by mock infection or *M. hyopneumoniae* infection. Furthermore, the mRNA expression of cytokines was determined in *M. hyopneumoniae*-infected cells. As expected, the mRNA of TRIF, MYD88 and TNF-α significantly decreased in NOD1 si2815 cells compared with that in the NC siRNA cells after *M. hyopneumoniae* challenge (**Figure 3**). Taken together, these results suggested that NOD1 silencing blocks the activation of pro-inflammatory cytokines triggered by *M. hyopneumoniae*.

### NOD1 Inhibits the Proliferation of *M. hyopneumoniae* During Infection

**Figure 4:**
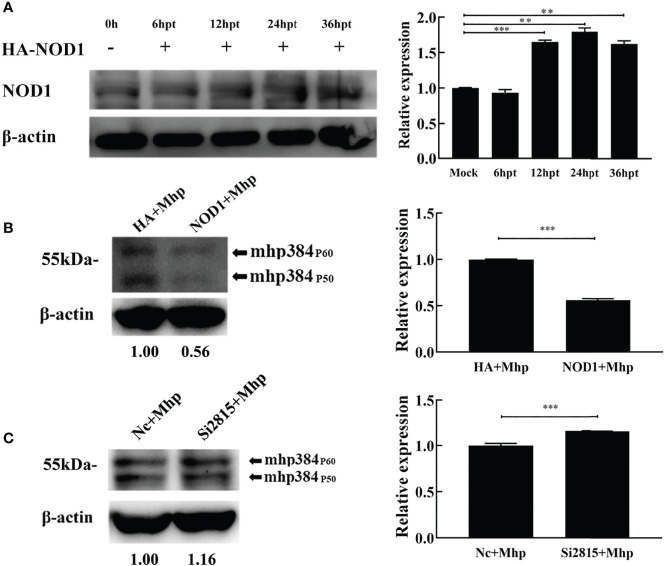
NOD1 inhibits *M. hyopneumoniae* replication during infection. **(A)** Alveolar macrophages were transfected with pCMV-HA-NOD1 plasmid or empty pCMV-HA vector. At 0, 6, 12, 24, or 36 hpt, the cells were lysed and subjected to western blotting analysis. Relative expression levels of NOD1 are presented as folds in the right panel. **(B)** Cells were transfected with pCMV-HA-NOD1 or pCMV-HA for 24 h, and then infected with *M. hyopneumoniae* (100 μL, 10^8^ CCU). **(C)** Cells were transfected with Nc siRNA or si2815 for 24 h, and then infected with M. hyopneumoniae (100 μL, 10^8^ CCU). Cells were collected at 24 hpi, and subjected to western blotting to determine the expression of *M. hyopneumoniae* mhp384. **P < 0.01, and ***P < 0.001 indicate statistically significant differences among different groups.

To assess the role of NOD1 in *M. hyopneumoniae* proliferation, we evaluated the yield of *M. hyopneumoniae* in PAM cells transfected with hemagglutinin (HA)-NOD1-expressing plasmid (**Figure 4A**). At 24 hpt, the transfected cells were infected with equal amounts of *M. hyopneumoniae*. The abundance of mhp384 protein from *M. hyopneumoniae*, which can indirectly indicate the amount of *M. hyopneumoniae*, was then determined at 24 hpi. The results demonstrated that overexpression of NOD1 significantly suppressed the proliferation of *M. hyopneumoniae* compared with the HA infected group (**Figure 4B**). In contrast, the abundance of mhp384 was slightly increased in NOD1 knockdown cells compared with that in the NC siRNA cells after *M. hyopneumoniae* challenge (**Figure 4C**).

### *M. hyopneumoniae* Adheres to Alveolar Macrophages and is Co-Localized With NOD1

**Figure 5:**
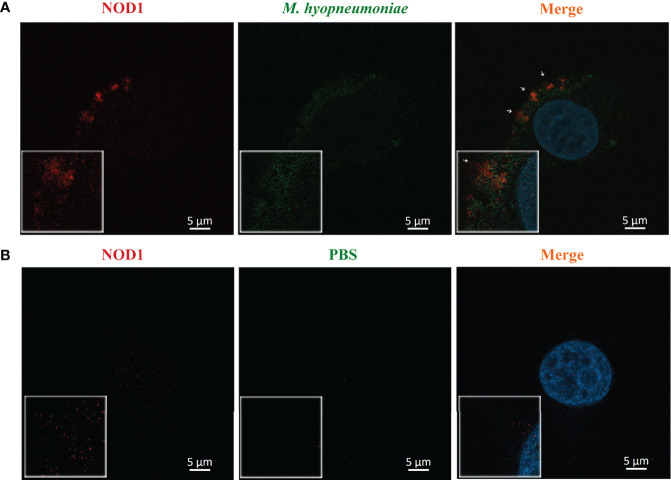
Confocal laser scanning micrograph for the adhesion of *M. hyopneumoniae* cells to alveolar macrophages. Alveolar macrophages were treated with *M. hyopneumoniae* **(A)** and PBS **(B)**, respectively. *M. hyopneumoniae* cells were labeled with CFDA-SE (green); cell nuclei were stained with DAPI (blue); and NOD1 was labeled red by using mouse anti-NOD1 mAb and goat anti-mouse IgG-Cy3. *M. hyopneumoniae* cells were adhered to the edges of alveolar macrophages, presenting a merged yellow signal, where *M. hyopneumoniae* was co-localized with the cellular NOD1 (white arrows). Scale bars = 5 µm.

The adhesion of *M. hyopneumoniae* to alveolar macrophages was investigated through fluorescence microscopy. Briefly, the host NOD1 was stained red by mouse anti-NOD1 mAb and goat anti-mouse IgG-Cy3, while the *M. hyopneumoniae* cells were labeled with CFDA-SE (green). As shown in **Figure 5**, *M. hyopneumoniae* infection up-regulated the expression of NOD1 compared with the mock infection control. *M. hyopneumoniae* cells were adhered and ingested into the alveolar macrophages and localized to regions enriched in NOD1, as indicated by the merged yellow signal. These observations suggested an intimate association between *M. hyopneumoniae* and host NOD1.

### mhp390 (P68) Mediates Cell Adhesion

**Figure 6:**
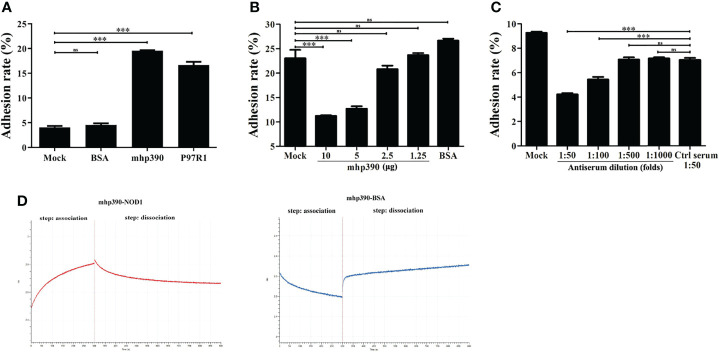
mhp390 mediates *M. hyopneumoniae* adhesion to alveolar macrophages and interacts with NOD1. **(A)** Adhesion of rMhp390 to alveolar macrophages detected by flow cytometry. **(B)** The adhesion of *M. hyopneumoniae* to alveolar macrophages was inhibited by rMhp390. The cells were incubated with different concentrations of rMhp390 before infection. BSA (10 μg/mL) and 1 mL of RPMI 1640 alone were used as the negative and blank controls, respectively. **(C)** The adhesion of *M. hyopneumoniae* to alveolar macrophages was inhibited by anti-rMhp390 serum. *M. hyopneumoniae* was incubated with anti-rMhp390 serum diluted from 1:50 to 1:1000 before infection. The mixed negative serum (unimmunized rabbit serum, 1:50 dilution) was used as the negative control. ***P < 0.001 indicate statistically significant differences among different groups, and ns represents no difference. **(D)** Kinetic analysis of the affinity between mhp390 and NOD1 through bio-layer interferometry.

The binding ability of mhp390 to alveolar macrophages was determined by using flow cytometry. Briefly, PAM cells were seeded in 24-well plates, and then infected with rMhp390. The unbound protein was removed, and the bound rMhp390 was labeled green through anti His-tag mouse mAb and goat anti-mouse IgG-FITC. *M. hyopneumoniae* adhesin P97R1 and BSA were used as positive and negative control, respectively. The results demonstrated that mhp390 has a strong adhesive capacity to alveolar macrophages (**Figure 6A**). In the positive control group, P97R1 showed a 3.65-fold adherence activity relative that of the mock group.

We further investigated the role of mhp390 in *M. hyopneumoniae* adhesion through flow cytometry. The adhesion of *M. hyopneumoniae* to PAM cells was first tested. Briefly, *M. hyopneumoniae* was cultured in a 5% CO_2_ atmosphere at 37°C for 48 h and fluorescently labeled by incubation with CFDA-SE. Then, the bacteria were washed to remove excess CFDA-SE. PAM cells (10^6^) were infected with fluorescently labeled *M. hyopneumoniae* at 37°C for 30 min at a multiplicity of infection (MOI) of 1:100. The cells were evaluated with flow cytometry and the adhesion ability of *M. hyopneumoniae* was evaluated based on the fluorescence intensity of PAM cells.

Subsequently, the inhibitory effect of both rMhp390 and antiserum on the adhesion of *M. hyopneumoniae* to PAM cells was assayed. To check the binding inhibition effect of rMhp390, *M. hyopneumoniae* was pre-blocked with two-fold serial dilution of rMhp390. The adhesion rate between the *M. hyopneumoniae* and alveolar macrophages was determined. With increasing rMhp390 concentration, more significant and dose-dependent inhibition of the binding between *M. hyopneumoniae* and alveolar macrophage was observed (**Figure 6B**). In contrast, no significant inhibition effect was found in the BSA and mock groups. As shown in **Figure 6C**, the binding of *M. hyopneumoniae* to PAM cells was effectively inhibited by antiserum directed against rMhp390 in a dose-dependent manner, whereas the negative serum from mock-immunized rabbit did not affect *M. hyopneumoniae* binding.

### mhp390 (P68) Interacts With NOD1

To gain more insights into the mechanism for the activation of NOD1 signaling by *M. hyopneumoniae*, we immobilized rMhp390 on biosensors and analyzed its interaction with rNOD1 through biolayer interferometry (BLI, Octet. RED 96e). As shown in the association and dissociation kinetic chart (**Figure 6D**), rNOD1 was rapidly bound to but slowly dissociated from SA-biosensors coated with rMhp390. rMhp390 bound rNOD1 with a high affinity (K_D_ = 8.14 μM). In the negative control group, no interaction was observed, with no value of K_D_.

To determine whether NOD1 plays a role in autophagy induction upon *M. hyopneumoniae* challenge, PAM cells were transfected with NOD1 siRNA or NC negative control siRNA before *M. hyopneumoniae* infection. Cell lysates were incubated with anti-LC3 antibody and analyzed through Western blotting. The LC3II/I ratio was determined by using ImageJ. As shown in **Figure S1**, *M. hyopneumoniae* activated the autophagy of PAM cells, whereas NOD1 silencing did not impede the autophagy induction. The results suggested that *M. hyopneumoniae* may induce autophagy through some other pathways instead of the NOD1 signaling pathway.

## Discussion

It has been widely recognized that NLRs sense PAMPs and play a crucial role in host immune response against viral and bacterial infection (Kumar et al., 2013; Oviedo-Boyso et al., 2014). In this study, we for the first time investigated the role of NOD1 in *M. hyopneumoniae* infection and provided evidence that *M. hyopneumoniae* activates the NOD1-RIP2 pathway to induce pro-inflammatory cytokine production.

Interstitial pneumonia is one of the most obvious clinical symptoms of *M. hyopneumoniae* infection, indicating that inflammatory response plays a critical role in the pathogenesis of *M. hyopneumoniae* (Lee et al., 2021). During the colonization of *M. hyopneumoniae* in the lower respiratory tract, a strong inflammatory response would be stimulated by inducing macrophages to release high levels of cytokines such as TNF-α, IL-1, IL-6, and IL-8 (Damte et al., 2011; Hwang et al., 2011). Large amounts of pro-inflammatory cytokines and high cytotoxic and phagocytic activities of macrophages are responsible for the development of lung tissue injury (Garcia-Morante et al., 2016). These innate immune responses can only be activated when the innate immune system can recognize the invading *Mycoplasma* through particular receptor-ligand interaction. Previous studies have documented that a heterodimer derived from TLR2 and TLR6 can recognize bacterial lipoproteins and plays an important role in sensing *Mycoplasma* (Takeda et al., 2002; Muneta et al., 2003; Takeuchi and Akira, 2007). It has been reported that the TLR1/TLR6 heterodimer is involved in sensing the dipalmitoylated lipoproteins from *M. pneumonia* (Shimizu et al., 2005). *M. pneumoniae* can induce inflammatory responses through TLR4 and mediate the toxin-dependent activation of inflammasome through several NLRs such as NLRP3 (Shimizu, 2016), resulting in the release of IL-18 and IL-1β (Khare et al., 2012; Segovia et al., 2018). TLR2 and TLR6 are also important in the recognition of *M. hyopneumoniae*, and blocking of these receptors would reduce the production of TNF-α in porcine alveolar macrophages (Okusawa et al., 2004).

**Figure 7:**
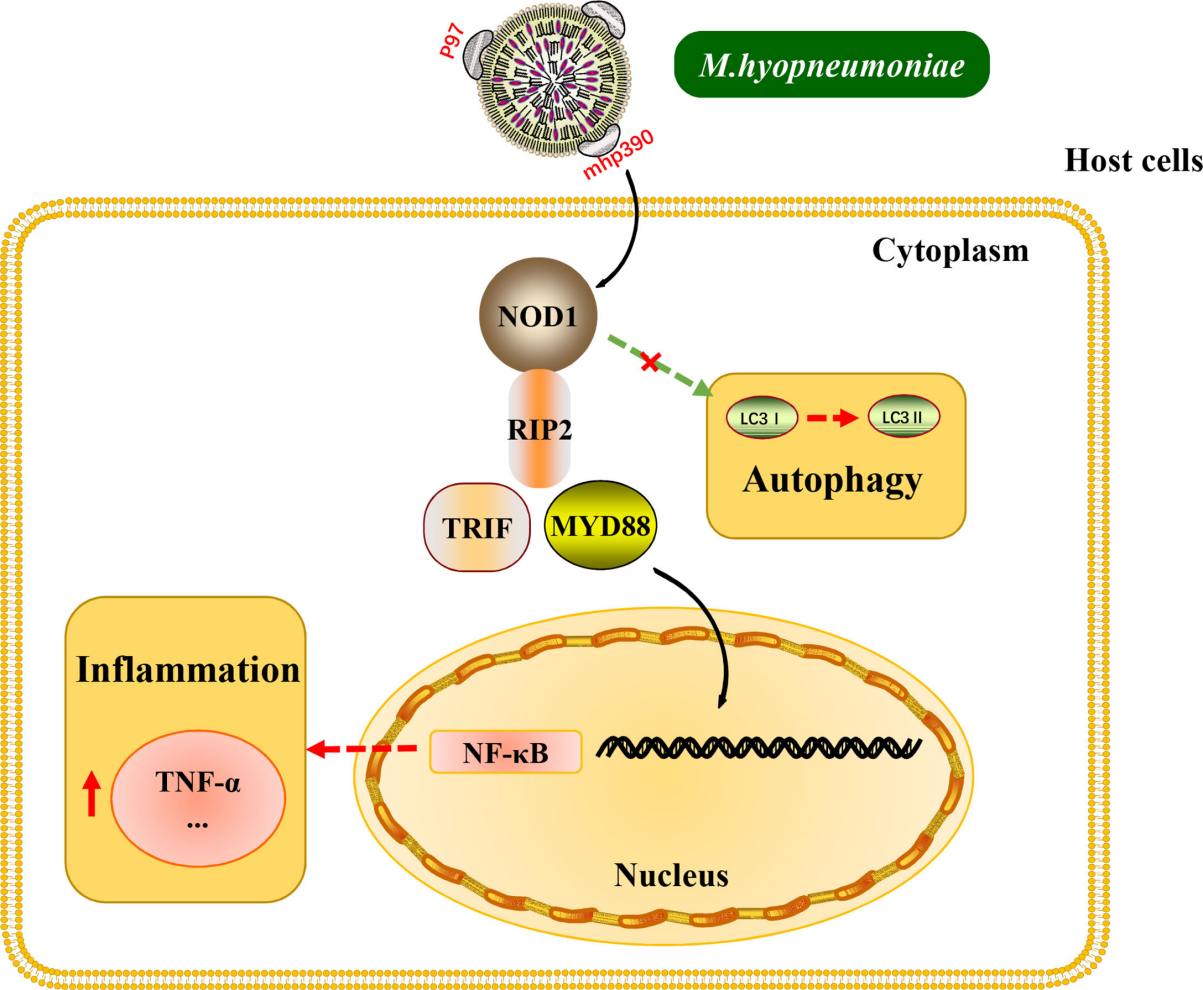
Schematic representation of the NOD1-RIP2 signaling pathway in *M. hyopneumoniae* invasion of alveolar macrophages.

Interestingly, the above inflammatory responses and the release of cytokines induced by *M. hyopneumoniae* are all dependent on TLR receptors. The results of the present study provide new insights into the mechanism for *M. hyopneumoniae* to activate inflammatory response through the NOD1 receptor (**Figure 7**). Besides, it is worth noting that *M. hyopneumoniae* should utilize some of its own proteins to interact with the NOD1 receptor to activate the subsequent signal cascade. These interesting issues have been studied by our laboratory. The results of NOD1 expression kinetics revealed that *M. hyopneumoniae* infection could increase the abundance of NOD1 in the host at both protein and mRNA levels. By confocal laser scanning microscopy, we found that *M. hyopneumoniae* cells were adhered to the edges of alveolar macrophages and co-localized with cellular NOD1. The role of NOD1 in the triggering of inflammatory responses by *M. hyopneumoniae* was also assessed. NOD1 silencing blocked the activation of pro-inflammatory cytokines triggered by *M. hyopneumoniae*, such as TRIF, MYD88, and TNF-α. The importance of the NOD-RIP2 pathway in accelerating secondary bacterial infection has been reported for several viruses (Vissers et al., 2012). It has been documented that murine norovirus-1 activates NOD1 and NOD2 signaling, which can accelerate the secondary *E. coli* infection (Kim et al., 2011). Besides, porcine reproductive and respiratory syndrome virus (PRRSV) infection can augment the NOD2-RIP2 signaling pathway to induce pro-inflammatory response. *M. hyopneumoniae* frequently co-infects the host with PRRSV, *Pasteurella multocida*, *Actinobacilus pleuropneumoniae*, and *Haemophilus parasuis*, contributing to the respiratory outbreak (Weissenbacher-Lang et al., 2016; Saade et al., 2020). Since NOD receptors can sense foreign pathogens and are responsible for triggering host inflammatory response, the pro-inflammatory cytokines mass produced *via* these pathways might result in more severe inflammation and expedite the development of lung lesions.

NOD receptors have been reported to play a variety role in the replication of different viruses. For instance, NOD1 activation is known to up-regulate antiviral response and suppress human cytomegalovirus replication (Fan et al., 2016). Moreover, NOD2 can inhibit the replication of respiratory syncytial virus, influenza virus and foot-and-mouth disease virus, but facilitates that of coxsackievirus B3 (Vissers et al., 2012; Le Bel and Gosselin, 2015; Liu et al., 2019a). Interestingly, we found that NOD1 had antibacterial activity upon *M. hyopneumoniae* infection. Interference of NOD1 blocked the activation of proinflammatory cytokines, whereas NOD1 overexpression significantly suppressed *M. hyopneumoniae* proliferation. We speculate that this antibacterial activity may be ascribed to the activation of inflammatory responses by NOD1 signaling.

*M. hyopneumoniae* mhp390 is a multifunctional protein (Liu et al., 2019b). Our previous study has shown that it is a new cilia adhesin with a strong binding ability for porcine respiratory cilia. Also, mhp390 could induce significant apoptosis of host immune cells. In addition, mhp390 stimulation was found to result in high levels of pro-inflammatory cytokines (Liu et al., 2019b). In this study, *M. hyopneumoniae* was observed to bind to alveolar macrophages and be co-localized with NOD1. The flow cytometry data confirmed that mhp390 mediates the adhesion of *M. hyopneumoniae* cells to alveolar macrophages. Furthermore, a strong interaction was found between host NOD1 and mhp390. Taken together, these findings suggest that mhp390 plays a crucial role in the interaction between *M. hyopneumoniae* and host cells. However, the specific domain responsible for this interaction remains unknown and needs further investigation.

In conclusion, our results demonstrate that *M. hyopneumoniae* activates the NOD1-RIP2 pathway and is co-localized with host NOD1 during infection. *M. hyopneumoniae* also employs NOD1 to induce pro-inflammatory response by influencing the TRIF and MYD88 pathway, thereby resulting in enhanced antibacterial activity of the host. Moreover, we for the first time described the interaction between *M. hyopneumoniae* mhp390 and NOD1 receptor, and the results indicated that mhp390 and NOD1 are possibly involved in the recognition of *M. hyopneumoniae.* These findings enrich our understanding of the crosstalk between pattern recognition receptors and *M. hyopneumoniae* and the cellular antibacterial immunity against *M. hyopneumoniae.*

## Data Availability Statement

The original contributions presented in the study are included in the article/**Supplementary Material**. Further inquiries can be directed to the corresponding authors.

## Author Contributions

WL, PS and YT conceived and designed the experiments. PJ, KY, FY, CL and ZL performed the experiments. WL, TG, DZ and QS analyzed the data. WL wrote the paper. All authors contributed to the article and approved the submitted version.

## Funding

This work was supported by The National Natural Sciences Foundation of China (31502069), Hubei Province Innovation Center of Agricultural Sciences and Technology (2019-620-000-001-17), Natural Science Foundation of Anhui (N0.1708085MC83), Natural Science Foundation of Tianjin (19JCQNJC13700), Research Project of Tianjin Education Commission (2018KJ185).

## Conflict of Interest

The authors declare that the research was conducted in the absence of any commercial or financial relationships that could be construed as a potential conflict of interest.

## Publisher’s Note

All claims expressed in this article are solely those of the authors and do not necessarily represent those of their affiliated organizations, or those of the publisher, the editors and the reviewers. Any product that may be evaluated in this article, or claim that may be made by its manufacturer, is not guaranteed or endorsed by the publisher.

## Supplementary Material

Supplementary Figure 1 NOD1 may not participate in the induction of autophagy by *M. hyopneumoniae*. **(A)** Alveolar macrophages were transfected with NOD1 siRNA or NC siRNA for 24 h, and the cells were then mock infected or infected with *M. hyopneumoniae* (100 μL, 10^9^ CCU). The cells were collected at 9 hpi and subjected to Western blotting by using anti-LC3 polyclonal antibody. **(B)** The LC3II/I ratio is shown as folds in the right panel.

Supplementary Table 1 Oligonucleotide primer sequences used in this study for gene amplification and qRT-PCR.
