# Supplementary figures and images for: Mycoplasma hyopneumoniae Infection Activates the NOD1 Signaling Pathway to Modulate Inflammation
Source: Front Cell Infect Microbiol. 2022 Jul 8;12:927840. doi: 10.3389/fcimb.2022.927840 (PMC9304885; doi:10.3389/fcimb.2022.927840)

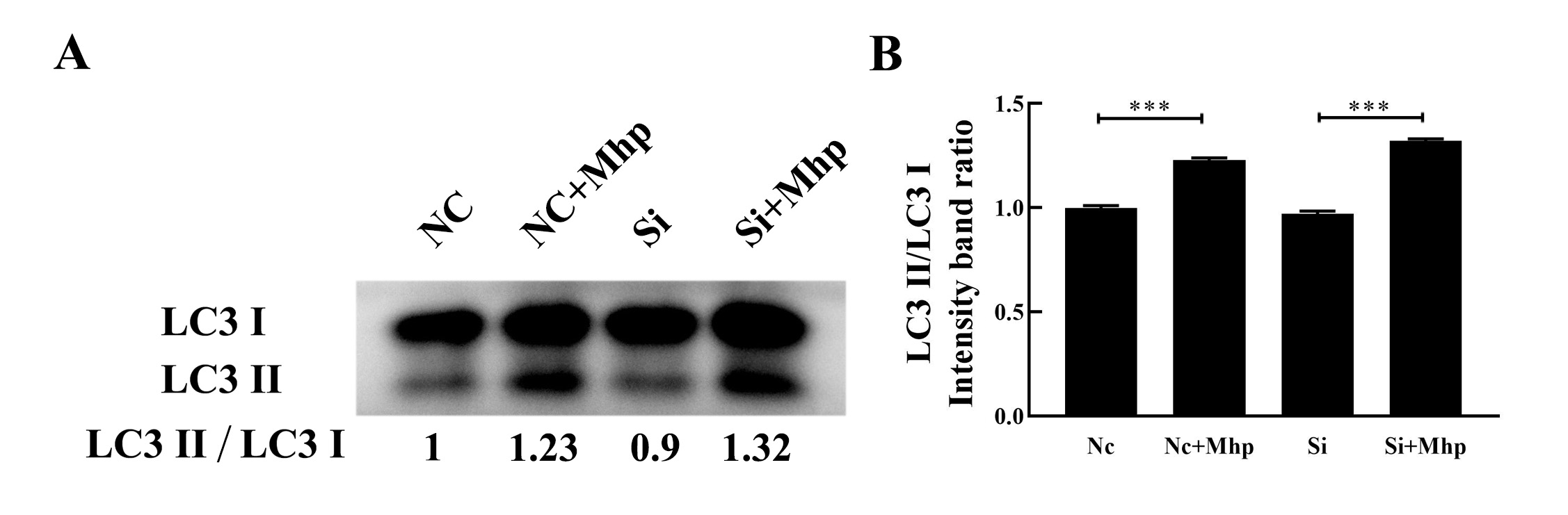

Supplement: Supplementary Figure 1 — NOD1 may not participate in the induction of autophagy by M. hyopneumoniae. (A) Alveolar macrophages were transfected with NOD1 siRNA or NC siRNA for 24 h, and the cells were then mock infected or infected with M. hyopneumoniae (100 μL, 109 CCU). The cells were collected at 9 hpi and subjected to Western blotting by using anti-LC3 polyclonal antibody. (B) The LC3II/I ratio is shown as folds in the right panel. [file Image_1.tif]
